# Clade 2.3.4.4b H5N1 highly pathogenic avian influenza detected in wild bird feces, Eastern Mongolia, 2024

**DOI:** 10.4142/jvs.25274

**Published:** 2026-05-26

**Authors:** Se-Hee An, Temuulen Myagmarsuren, Gyeong-Beom Heo, Hwanseok Jeong, Yong-Myung Kang, Urankhaich Dondog, Unurjavkhlan Tsogbadrakh, Youn-Jeong Lee, Erdene-Ochir Tseren-Ochir, Kwang-Nyeong Lee

**Affiliations:** 1 Avian Influenza Research & Diagnostic Division, Animal and Plant Quarantine Agency, Gimcheon 39660, Korea.; 2 Department of Infectious Disease and Microbiology, School of Veterinary Medicine, Mongolian University of Life Sciences, Ulaanbaatar 17024, Mongolia.; 3 College of Veterinary Medicine & Institute for Veterinary Biomedical Science, Kyungpook National University, Daegu 41566, Korea.

**Keywords:** Interregional dissemination, breeding and wintering grounds, whooper swan, phylogenomics, one health

## Abstract

**Importance:**

Despite ongoing intercontinental spread of clade 2.3.4.4b H5N1 highly pathogenic avian influenza (HPAI), surveillance data from Mongolia -- a critical junction between Central and East Asian migratory flyways -- remain scarce, leaving a gap in understanding how viruses move between northern breeding areas and southern wintering grounds.

**Objective:**

We aimed to detect and genetically characterize HPAI viruses in wild bird populations in Mongolia and assess their relationship with viruses circulating in East Asia.

**Methods:**

Active surveillance was conducted at Ganga Lake, Mongolia, in October 2024. A total of 352 wild bird fecal samples were collected and tested for AIVs. Viral isolates were subtyped, whole-genome sequenced, and phylogenetically analyzed. Host species was identified using c oxidase I barcoding.

**Results:**

Sixteen AIVs were isolated, including one clade 2.3.4.4b H5N1 HPAI virus (M9-3) from whooper swan feces. Genomic analysis revealed M9-3 had > 99% similarity with Korean and Japanese H5N1 isolates reported in late 2024 and undergone recent reassortment and disseminated through migratory flyways.

**Conclusions and Relevance:**

This is the first detection of clade 2.3.4.4b H5N1 in Eastern Mongolia in a fecal sample during late autumn. These findings underscore Mongolia's importance as a sentinel location for interregional HPAI dissemination.

## INTRODUCTION

Highly pathogenic avian influenza (HPAI) H5 viruses have caused tremendous losses in the poultry industry since the emergence of the Gs/GD lineage H5 HPAI virus in 1996 in Guangdong Province, China [1]. That lineage diverged into clades 0 to 9, and clade 2 became the predominant clade with continuous evolution and expansion of subclades [2]. Among the subclades, subclade 2.3.4.4b H5N1 HPAI has become dominant and spread to various continents, including Europe, Asia, the Americas, and Africa. Viruses of this clade are associated with high mortality in some cases, affecting not only wild birds but also mammals, posing a significant public health threat [3].

In Mongolia, there have been several H5 HPAI outbreaks in clinically ill or dead wild birds that were returning to the northern territory in spring, involving clade 2.2 H5N1 in 2005–2006, clade 2.3.2.1 H5N1 in 2009–2010 and clade 2.3.4.4 H5N6 in 2020 [4]. Genetically, these cases were closely related to the preceding ones reported on Xinjiang, Qinghai or Tibet, which bordered on the southwest of Mongolia [4,5]. Particularly, the clade 2.2 H5N1 HPAI virus was also genetically close to the viruses detected in Inner Mongolia, Liaoning Province, China, and Russia in October and November 2005, which surrounds eastern Mongolia [6,7]. Interestingly, all of these viruses were isolated from dead or ill wild birds captured in lakes from April to July after the spring migration of migratory birds, and no HPAI virus has been isolated from live wild birds to date even with continuing active surveillance on wild bird including fecal samples in Mongolia [4,5,6,8]. It was suggested that wild bird migration was the major cause of the Mongolian HPAI outbreaks and intercontinental spread as the viruses were genetically related to those detected along the migratory flyways and, to date, HPAI detections in Mongolia have been limited to wild birds with no confirmed cases in poultry (World Organisation for Animal Health [WOAH] World Animal Health Information System, accessed 2026-01-20) though the Mongolia’s poultry industry is relatively small (about 1,836,500 heads, National Statics Office of Mongolia, accessed 2026-01-20) compared to neighboring countries [5].

Since 2021, we have been conducting active surveillance annually in eight Mongolian lakes from May to October, which migratory birds use as summering sites or destinations for stopover, resting, foraging and congregation during their migration periods (Supplementary Table 1) [8]. These lakes sit at the two major flyways, the East-Asian Australasian flyway and the Central Asian flyway [9,10]. In this study, we isolated a clade 2.3.4.4b H5N1 HPAI virus from a fecal sample collected in Ganga Lake in Sukhbaatar province on 13 October 2024, and we determined the whole-genome sequences of the virus and compared them with the sequences of other HPAI viruses isolated in Korea and in the GISAID database.

## METHODS

### Surveillance and sample collection

Active surveillance was conducted at eight Mongolian lakes from May to October 2024 to monitor migratory birds along major flyways. A total of 352 wild bird fecal samples collected from Ganga Lake on 13 October 2024 were screened for avian influenza virus (AIV) using specific pathogen-free embryonated chicken eggs and a rapid antigen kit (Bionote, Korea) isolation in the BSL3 facility of the Institute of Veterinary Medicine in Mongolia as described in the WOAH terrestrial manual 3.3.4. (Supplementary Data 1). The RNA was subsequently sent to Animal and Plant Quarantine Agency for subtyping, pathotyping and further genetic analysis. In parallel, wild bird feces collected on 29 October 2024 in Pocheon, Korea and an oropharyngeal sample from HPAI infected broiler breeder farm on 17 November 2024 in Ganghwa, Korea were prepared for comparative genomic analysis.

### Molecular subtyping and host identification

AIV matrix, H5, and H7 genes were screened via quantitative reverse transcription polymerase chain reaction (qRT-PCR) using the VDx AIV qRT-PCR Ver 2.1 kit (Median Diagnostics, Korea). Subtyping was conducted via in-house hemagglutinin (HA) and neuraminidase (NA) end-point RT-PCRs and the HA cleavage site of positive samples was determined by Sanger sequencing (Macrogen, Korea). For host species identification, total DNA and RNA were extracted from virus-positive feces, and the mitochondrial cytochrome c oxidase I (COI) gene was amplified and analyzed using the BOLD system database (Supplementary Data 1) [11].

### Whole-genome sequencing and phylogenomics

Eight viral gene segments were amplified using a one-step RT-PCR framework (Supplementary Data 1) [12,13]. Sequencing libraries were prepared and sequenced on the Oxford Nanopore MinION platform, and consensus genomes were generated. To infer genetic origins, maximum likelihood phylogenetic trees were constructed using RAxML on XSEDE, incorporating the top 100 BLAST hits for each gene segment retrieved from the GISAID database (Supplementary Data 1) [14].

## RESULTS

A total of 352 wild bird fecal samples were collected from Ganga Lake (45.3049042, 113.8076523) on October 13, 2024 (Supplementary Table 1). One of them, A/wild bird/Mongolia/M9-3/2024 (EPI_ISL_19825828), referred to as M9-3, was determined to be a clade 2.3.4.4b H5N1 HPAI virus through RT-PCR, Sanger sequencing of the H5 cleavage site and whole-genome sequencing via the Oxford Nanopore platform. The others were subtyped as eight H3N8, two H1N1, two H1N2, two H12N5 and one H7N2 AIV of low pathogenicity.

**Fig. 1:**
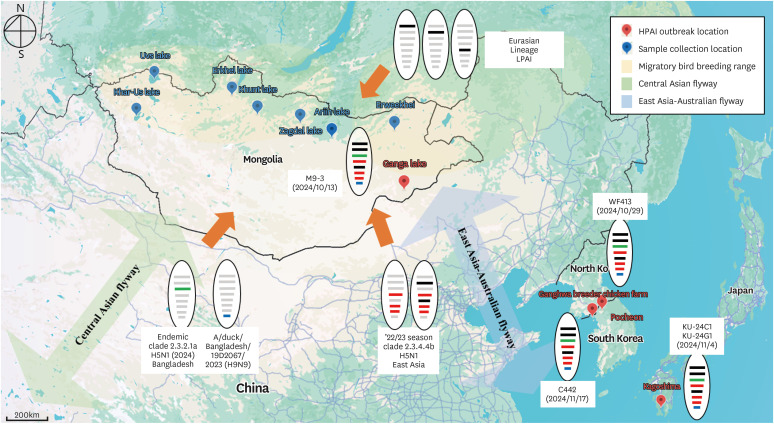
Locations of wild bird surveillance sites and clade 2.3.4.4b H5N1 HPAI outbreak sites in East Asia, autumn 2024. Eight Mongolian lakes (blue map pins) within the migratory bird breeding range were monitored from May to October. HPAI outbreak locations in Mongolia, Korea, and Japan are indicated by red map pins. The Central Asian flyway is shown by a green arrow, and the East Asia -Australasian flyway by blue arrows. Stacked bar icons beside each location show the genomic segment composition: segments with high nucleotide similarity to M9-3 are shown in the same color as M9-3, and dissimilar segments are shown in gray. The satellite base map was obtained from Google Earth (https://earth.google.com). HPAI, highly pathogenic avian influenza; LPAI, low-pathogenic avian influenza.

Through mitochondrial gene cytochrome COI analysis of the single fecal sample from which M9-3 was isolated, the bird species was specified as *Cygnus cygnus* (whooper swan) with 99.84% similarity and 100% species-level assignment confidence in the BOLD system and no sequences corresponding to other species detected [11]. In regard to this, flocks of whooper swan and swan goose were observed by the research team who had visited Ganga Lake to collect fecal samples in October 2024 (Fig. 1).

M9-3 has the multiple basic amino acids around the cleavage site, PLREKRRKR/GLF, and its whole genomic sequence is almost identical to the viruses isolated from Korean HPAI outbreaks during 2024/2025 winter season; the genotype of these viruses had not been reported prior to the 2024/2025 season, but it became one of the predominant genotypes among the several genotypes observed in poultry and wild birds of Korea in 2024/2025 winter season (data not shown). Two representative Korean isolates, A/wild bird/Korea/WF413/2024 (EPI_ISL_19825819) obtained from wild bird feces and A/Chicken/Korea/C442/2024 (EPI_ISL_19825820) from a breeder chicken farm of that genogroup detected at the earliest phase of the outbreak period of 2024/2025 winter season were selected for comparative genomic analyses. In addition, M9-3 had the highest nucleotide sequence identities at each gene segment level to the isolates from the environmental samples collected in Kagoshima, Japan, on 4 November 2024 in the GISAID database: A/environment/Kagoshima/KU-24C1/2024 (EPI_ISL_19534183), A/environment/Kagoshima/KU-24G1/2024 (EPI_ISL_19534185), and A/environment/Kagoshima/KU-24-B4/2024 (KU-24-B4, EPI_ISL_19624046). Compared to them, M9-3 had high nucleotide sequence identities at each gene segment level, ranging from 99.40%–99.91%, however, NP gene of KU-24-B4 had lower identity, 93.80%, to M9-3 (Table 1).

**Table 1:** The viruses sharing the highest homology with A/wild bird feces/Mongolia/M9-3/2024 (M9-3) and the nucleotide sequence identities of gene segments between the M9-3 and HPAI viruses isolated in Korea and Japan in the 2024–2025 winter season

Gene	Virus	Accession number	Range of % identity^a^
PB2	A/environment/Kagoshima/KU-24C1/2024 (H5N1)	EPI_ISL_19534183	99.65–99.82
PB1	A/environment/Kagoshima/KU-24-B4/2024 (H5N1)	EPI_ISL_19624046	99.74–99.82
PA	A/environment/Kagoshima/KU-24-B4/2024 (H5N1)	EPI_ISL_19624046	99.67–99.91
HA	A/environment/Kagoshima/KU-24-B4/2024 (H5N1)	EPI_ISL_19624046	99.47–99.82
NP	A/environment/Kagoshima/KU-24C1/2024 (H5N1)	EPI_ISL_19534183	99.67–99.87^b^
NA	A/environment/Kagoshima/KU-24-B4/2024 (H5N1)	EPI_ISL_19624046	99.43–99.86
M	A/environment/Kagoshima/KU-24-B4/2024 (H5N1)	EPI_ISL_19624046	99.80–99.90
NS	A/environment/Kagoshima/KU-24G1/2024 (H5N1)	EPI_ISL_19534185	99.40–99.76

HPAI, highly pathogenic avian influenza; PB, polymerase basic; PA, polymerase acidic; HA, hemagglutinin; NP, nucleoprotein; NA, neuraminidase; M, matrix; NS, nonstructural.

^a^Nucleotide sequence identity range between M9-3 (EPI_ISL_19825828) and five clade 2.3.4.4b H5N1 HPAI viruses from Korea (A/wild bird/Korea/WF413/2024 (EPI_ISL_19825819), A/Chicken/Korea/C442/2024 (EPI_ISL_19825820)) and Japan (A/environment/Kagoshima/KU-24C1/2024 (EPI_ISL_19534183), A/environment/Kagoshima/KU-24G1/2024 (EPI_ISL_19534185), and A/environment/Kagoshima/KU-24-B4/2024 (EPI_ISL_19624046)).

^b^A/environment/Kagoshima/KU-24-B4/2024 shared 93.80% nucleotide similarity with M9-3 for NP gene.

In addition to comparing M9-3 with contemporary H5N1 viruses detected in Mongolia, Korea, and Japan during the 2024/2025 winter season (Table 1), we also analyzed earlier viruses reported before the first detection in Mongolia on 13 October 2024 (Table 2). For this purpose, the viruses showing the highest sequence identity to each gene segment among the pre-October isolates were summarized separately in Table 2, allowing us to infer the possible genetic origins of the newly identified genotype. The M9-3 showed high genomic similarities with the viruses of clade 2.3.4.4b H5N1 predominant in 2022/2023 in East Asian countries for the HA, NA, and matrix genes; for these genes, it had nucleotide identities, 98.83%, 99.22%, and 99.59%, respectively, against A/Mandarin duck/Korea/WA496/2022, representing the preceding predominant genotype in 2022/2023 winter season (Supplementary Fig. 1, Table 2) [15,16]. Five other genes (PB2, PB1, PA, NP, and NS) of M9-3 were clustered with genes of Eurasian lineage low-pathogenic avian influenza (LPAI) viruses of various subtypes or some H5N1 viruses of clade 2.4.4.4b or clade 2.3.2.1a recently isolated in Asia; in particular, the PB1 and NP genes of M9-3 were closely related to the A/eagle/Korea/22WC464/2023 isolate of the clade 2.3.4.4b H5N1 which were detected in Korea in the 2022/2023 winter season, with 98.72 and 98.66 identities, respectively (Supplementary Fig. 1, Table 2) [15], and the PA and NS genes were closest to those of endemic clade 2.3.2.1a H5N1 and LPAI viruses isolated in Bangladesh during 2023–2024 (Supplementary Fig. 1, Table 2).

**Table 2:** The virus with the highest homology with A/wild bird feces/Mongolia/M9-3/2024 (M9-3) before the 2024–2025 winter season and the nucleotide sequence identities of the gene segments

Gene	Virus	Accession number	% Identity^a^
PB2	A/Spot-billed duck/South Korea/KNU2022-73/2022 (H4N6)	EPI_ISL_19109827	98.82
PB1	A/spot billed duck/Korea/KNU-30/2022 (H1N1)	EPI_ISL_18698826	98.94
PA	A/duck/Bangladesh/17-D-2338/2024 (H5N1)	EPI_ISL_19183994	99.21
HA	A/Mandarin duck/Korea/WA496/2022 (H5N1)	EPI_ISL_15647836	98.83
NP	A/eagle/Korea/22WC464/2023 (H5N1)	EPI_ISL_18245369	98.66
NA	A/hooded crane/Kagoshima/KU-134/2022 (H5N1)	EPI_ISL_18472686	99.36^b^
M	A/Mandarin duck/Korea/WA496/2022 (H5N1)	EPI_ISL_15647836	99.59
NS	A/duck/Bangladesh/19D2067/2023 (H9N9)	EPI_ISL_18983962	99.16

PB, polymerase basic; PA, polymerase acidic; HA, hemagglutinin; NP, nucleoprotein; NA, neuraminidase; M, matrix; NS, nonstructural.

^a^Nucleotide sequence identity between M9-3 and the virus with the highest homology.

^b^The NA gene of A/Mandarin duck/Korea/WA496/2022 (H5N1) showed 99.22% sequence identity with that of M9-3.

## DISCUSSION

The genotype of the clade 2.3.4.4b H5N1 virus detected in Mongolia (M9-3) represents a novel genomic constellation that has not been reported previously in Mongolia, Korea, or Japan. In light of this, we suggest that the M9-3 might have been generated by genetic reassortment of AIVs introduced to the northern breeding sites via various flyways, and the Central Asian flyway, which stretches from West Asia, India or Bangladesh northward through Mongolia to the northernmost grounds of Siberia, has been used for the northward movement of AIVs endemic in nonbreeding wintering grounds [17,18]. Especially, the Ganga lake and its surrounding wetlands, located in the southernmost area in Eastern Mongolia, are composed of various small lakes, streams, and natural springs and Ganga lake is well known for congregation of large swan and goose populations (Fig. 1). The virus was detected on 13 October 2024 in Ganga lake, earlier than the first genetically related viruses identified in Korea and Japan, which were collected from late October to mid-November 2024. This temporal pattern aligns with the well-documented southward migration of whooper swans and swan geese from northern breeding areas toward East Asia during the autumn period [19]. Considering 1) the emergence of a previously unreported genotype, 2) the earlier detection in Mongolia, and 3) the established north-to-south migratory movements of the potential host species, the findings are most consistent with the introduction of newly reassorted viruses from northern breeding sites in Mongolia, followed by subsequent dissemination toward Korea and Japan. While alternative introduction routes cannot be completely excluded, the combined genomic and ecological evidence supports the likelihood of north-to-south movement along migratory flyways during the 2024 autumn season (Fig. 1).

This study describes a clade 2.3.4.4b H5N1 HPAI virus detected in fecal samples from Ganga Lake in eastern Mongolia just before autumn migration of wild birds in early October 2024. The virus shared high genetic identity with clade 2.3.4.4b H5N1 HPAI viruses in Korea and Japan detected at the end of October or in November of the same year, 2024. Thus, this provides convincing evidence that the viruses produced and circulated in breeding areas in Eurasia in summer are introduced into wintering grounds in East Asia. Therefore, strategic active surveillance at breeding or stopover sites just before bird migration in mid-October may serve an earlier warning indicator to provide crucial information about emerging viruses for national preparedness and response planning ahead of the winter season to countries along downstream migratory routes, including Korea. In addition, early warning information of HPAI capable of crossing species barriers is essential from a One health perspective, as timely detection in wildlife can help mitigate subsequent risks to poultry and human health.

## SUPPLEMENTARY MATERIALS

Supplementary Data 1 Supplementary methods

Supplementary Table 1 Surveillance results of wild bird fecal samples collected at Mongolian migratory breeding sites from May to October 2024

Supplementary Fig. 1 Maximum likelihood phylogenetic tree of eight gene segments. The branch length is shown for each root and node. Clade 2.3.4.4b H5N1 HPAI viruses isolated in Mongolia, Korea and Japan in 2024 are denoted with different node shapes and text colors: red stars represent Mongolian HPAI, orange triangles represent Korean HPAI, and green diamonds represent Japanese HPAI. Other viruses are colored according to their pathogenicity, clade and subtype, and the LPAI is labeled in black without a node shape. (A) PB2, (B) PB1, (C) Polymerase acidic, (D) Hemagglutinin, (E) Nucleoprotein, (F) Neuraminidase, (G) Matrix, (H) Nonstructural.
