# Supplementary material for: Clade 2.3.4.4b H5N1 highly pathogenic avian influenza detected in wild bird feces, Eastern Mongolia, 2024
Source: J Vet Sci. 2026 May 26;27(4):e38. doi: 10.4142/jvs.25274 (PMC13454792; doi:10.4142/jvs.25274)
Supplement: Supplementary file 1 [file jvs-27-e38-s001.doc]

**Supplementary methods**

**Detailed viral isolation protocols**

Fecal samples were suspended in phosphate-buffered saline (PBS) containing antibiotics to form a 10% (w/v) suspension. After centrifugation at 3,000 rpm for 5 min, 0.2mL of the supernatant was inoculated into five 10-day-old embryonated chicken eggs. Allantoic fluid harvested after 3–4 days of incubation at 37°C was tested using a rapid AIV antigen kit (Bionote, Korea) and a hemagglutination test. Viral RNA from isolated samples was extracted using the RNeasy Mini Kit (Qiagen, Germany). For Korean isolates, RNA was extracted using the NX-48 Viral NA Kit on a Nextractor NX-48 instrument (Genolution, Korea).

**Host DNA barcoding protocol**

Fecal samples were lysed using the Maxwell RSC simplyRNA Tissue Kit (Promega, USA). Total nucleic acids were extracted using the Maxwell RSC 48 instrument. The COI gene was amplified with Maxime PCR PreMix (iNtRON Biotechnology, Korea) using 1 μL each of AvesF and AvesR primers of 10 μM and 2 μL of DNA template in a 20μL reaction volume [11]. Thermal cycling conditions were: 5 min at 95°C; 35 cycles of 30 sec at 95°C, 1 min at 56°C, and 30 sec at 72°C; followed by a final extension for 7 min at 72°C.

**Nanopore whole-genome sequencing details**

One-step RT-PCR utilized the SuperScript III One-Step RT-PCR System with Platinum Taq DNA Polymerase (Invitrogen, USA) [12, 13]. The reaction mixture 50 μL included 10 μL RNA, 25 μL 2X buffer, 2μL enzyme mix, and specific primers: 0.5 μL CommonA-Uni12G (5’-GCCGGAGCTCTGCAGATATCAGCGAAAGCAGG-3’, 10 µM), 0.5 μL CommonA-Uni12 (5’-GCCAGAGCTCTGCAGATATCAGCAAAAGCAGG-3’, 10 µM), and 1μL CommonA-Uni13G (5’-GCCGGAGCTCTGCAGATATCAGTAGAAACAAGG-3’, 10 µM). Cycling profiles: 42°C for 15 min, 55°C for 15 min, 60°C for 5 min, 94°C for 2 min; 5 cycles at 94°C (30 sec), 45°C (30 sec, ramp 2.5°C/s), 68°C (5 min, ramp 0.5°C/s); 37 cycles at 94°C (30 sec), 55°C (30 sec), 68°C (5 min); and 68°C for 5 min. Amplicons were purified with 90 µL of Ampure XP SPRI Reagent (Beckman Coulter, USA) and quantified with a Qubit 4 fluorometer (Invitrogen). Libraries were prepared using the Native Barcoding Kit 96 V14 (SQK-NBD114.96) and sequenced on a MinION Mk1C platform using R10.4.1 Flow Cells (Oxford Nanopore Technology, UK). Base calling was performed via MinKNOW V.23.11.3, and consensus sequences were constructed using CLC Genomics Workbench 23.0.4.

**Phylogenetic analysis parameters**

The top 100 BLAST hits for each of the eight genes of the Mongolian isolate were downloaded from GISAID (as of 27 December 2024). Sequence numbers used: HA (274), NA (307), PB2 (624), PB1 (626), PA (626), NP (629), MP (619), and NS (622). Untranslated regions were removed, and protein-coding sequences were aligned using MAFFT with default parameters. Maximum likelihood trees were generated via RAxML on XSEDE (v8.2.12) via the CIPRES Science Gateway (https://www.phylo.org/) and visualized using CLC Main Workbench 20 [14].
